# Epidural Needle Extension through the Ligamentum Flavum Using the Standard versus the CompuFlo®-Assisted Loss of Resistance to Saline Technique: A Simulation Study

**DOI:** 10.1155/2020/9651627

**Published:** 2020-01-07

**Authors:** E. Capogna, A. Coccoluto, M. Velardo

**Affiliations:** European School of Obstetric Anesthesia, Maternal Neonatal Simulation Centre, Rome, Italy

## Abstract

**Background:**

The CompuFlo® epidural system has been recently introduced and validated as an objective and sensible tool to detect the epidural space. We aimed to verify whether the high sensitivity of the instrument may help the anesthesiologist to identify the epidural space very early, limiting the extension of the Tuohy needle into the epidural space.

**Methods:**

In this prospective, simulation study, we evaluated the Tuohy needle extension through a simulated ligamentum flavum during the epidural procedure performed by 52 expert anesthesiologists by using the CompuFlo® epidural instrument or their standard loss of resistance to saline technique (LORT).

**Results:**

The mean (SD) needle extension length was 3.90 (3.71) mm in the standard technique group and 0.68 (0.46) mm in the CompuFlo® group (*P* < 000001). The extremely reduced variability of the data in the CompuFlo® group (*F* test 0.01) made the results obtained with this instrument highly predictable.

**Conclusions:**

Puncturing high-resistance material that simulated the ligamentum flavum, the use of CompuFlo® has determined the arrest of the needle more precociously when compared with the traditional LORT.

## 1. Introduction

Accidental dural puncture (ADP) is particularly troublesome in the obstetric population because over half of all patients who experience ADP with epidural needles will eventually develop postdural headache [1].

Unfortunately, the overall quality of evidence for preventive measures is generally weak [2], and therefore good knowledge of lumbar anatomy together with a carefully performed technique are extremely important.

The thickness of the epidural space, from the internal surface of the ligamentum flavum to the external surface of the dural sac, has been reported to vary from 6 to 13 mm [3] which usually represents a sufficient length of space to safely stop the advancement of the Tuohy needle once it has entered the epidural space before it penetrates the dural sheath.

Even if it has not been demonstrated, it sounds reasonable that as the bevel of a Tuohy needle is no longer than a few millimetres, the sooner the physician stops the advancement of the needle once it has entered the epidural space (therefore, less epidural needle coming out of the ligamentum flavum), the less likely an inadvertent dural puncture is to occur.

In order to improve the epidural technique, many systems have been described. All prior devices [4] such as the epidrum or episure have a mechanical design which is unable to distinguish between a true-loss of resistance (LOR) and a false-LOR. Lechner et al. [5, 6] used continuous infusion of normal saline by an infusion pump to generate an acoustic signal and a continuous pressure reading while performing epidural block. However, the infusion pump used was unable to measure or control the pressure itself. New pressure based tools have been recently described [7–9], but unfortunately their use is confined to animal and experimental fields and the tools are not available on the market yet.

Epidural space identification by using continuous, quantitative, real-time, needle-tip pressure measurement (CompuFlo® epidural computer-controlled anesthesia system instrument, Milestone Scientific, Inc., Livingston, New Jersey, USA) has been recently introduced and has been demonstrated to be objective, precise, reliable, and highly sensitive in clinical practice [10–12].

We hypothesized that the CompuFlo® epidural system might help the anesthesiologist to identify the epidural space very precociously, most likely limiting the extension of the Tuohy needle into the ligamentum flavum and therefore, in theory, reducing the risk of ADP.

In this prospective, the simulation study, we evaluated the Tuohy needle extension through a simulated ligamentum flavum during the epidural procedure performed by expert anesthesiologists by using the CompuFlo® Epidural instrument or their standard loss of resistance to saline technique.

## 2. Method

Ethical approval was sought and was waived by the Local Research Ethics Committee because they deemed it not necessary as no patients were involved in the study.

After having obtained their informed consent, we enrolled 52 expert obstetric anesthesiologists who had never previously used the CompuFlo® Epidural instrument.

All the physicians had more than 10 years of expertise in obstetric epidural anesthesia.

**Figure 1:**
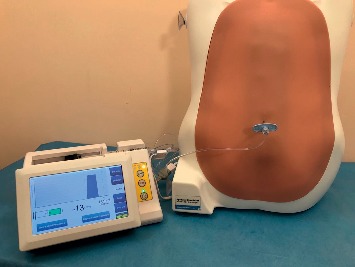
Experimental setting: the CompuFlo® and the epidural simulator used.

For the purpose of the study, we used the following materials: a 16G Tuohy needle (Portex, Smiths Medical), a standard, commercially available LOR insert-kit (3B Scientific® XP61-002 Kit) of an epidural simulator (3B Scientific® Epidural and Spinal Injection Trainer P61) which has been designed to mimic the resistance of the ligamentum flavum, and a CompuFlo® epidural instrument (Figure 1).

The CompuFlo® epidural instrument is a computer-controlled drug delivery system capable of distinguishing different tissue types by providing continuously real-time “exit-pressure” data at the needle tip and that has been validated as a useful tool in detecting the epidural space [10–12].

This instrument uses the continuous real-time pressure sensing technology, an algorithm to determine the pressure at the tip of the needle via a continuous fluid path. The pressure recorded at the tip of the needle acts for feedback and controller to the system, thus regulating the electromechanical motor which controls the flow rate and the fluid dispensed by the system. An audible feedback and a visual graphic of exit-pressure are provided to the physician to focus on the injection site.

The device is supplied with an Epidural Disposable Kit, which includes a 20 mL syringe, 48 inch tubing set, a pressure sensor, and an ID Adapter Key.

After having received instruction on the use of the CompuFlo® instrument and familiarized with the epidural simulator, the participants were randomized to which technique they did first (standard or CompuFlo®-assisted technique).

When using the standard technique, the participants were asked to connect the Tuohy needle to an epidural LOR syringe (Portex, Smiths Medical) filled with 5 mL of saline and carry out the epidural block with the loss of resistance technique in the same way they usually perform it.

When using the CompuFlo®-assisted technique, the entry of the needle into the ligamentum flavum (simulator's “high resistance area”) was indicated by a great increase in pressure on the visual display with a simultaneous increase of the pitch of the audible tone, while the entry of the needle into the epidural space resulted in a brisk drop in pressure and a distinct fall in the tone of the audio output. A drop in pressure sustained for more than 5 seconds was deemed to be consistent with entry into the epidural space [4–6].

In both groups, the physicians were asked to immediately stop the needle when the resistance was deemed to be lost.

**Figure 2:**
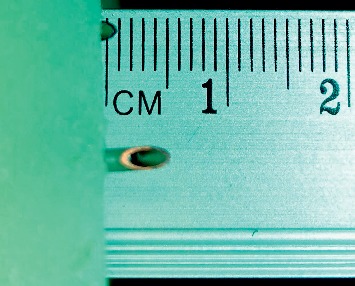
Magnification of the inner portion of the epidural simulator to show the method of measuring the extent of the epidural needle in the epidural space. In the study, each measurement was performed after each individual test by an independent observer.

The needles introduced into the LOR insert kit (ligamentum flavum) were kept in place, and the distance the needle tip had extended beyond its internal surface was measured by an independent observer using a mini 0.5 mm resolution precision stainless steel ruler (Starrett Precision Tools, Athol, MA, USA), blinded as to whom and how the procedure had been performed (Figure 2).

The primary aim of the study was to evaluate the differences in Tuohy needle extension through a simulated ligamentum flavum between the two groups. The power analysis was set to observe at least a 30% difference between the groups and required a sample size of 50 observations to set 80% test power and a 95% significance level. Unpaired *T*-test and ANOVA were used to evaluate the differences between the two groups.

## 3. Results

**Figure 3:**
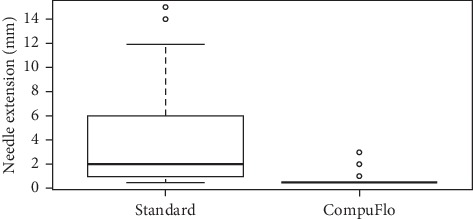
Box plot of the Tuohy needle extension (mm) through a simulated ligamentum flavum during the epidural procedure with the standard technique or with the CompuFlo®-assisted technique.

All the physicians completed the task. The mean (SD) needle extension length was 3.90 (3.71) mm in the standard technique group and 0.68 (0.46) mm in the CompuFlo® group (*P* < 0.00001). The box plot is reported in Figure 3 which emphasizes the great difference in the confidence intervals between the groups that, in turn, suggests an extremely reduced variability of the data in the CompuFlo® group, making the results obtained with this instrument highly predictable. This was also confirmed by the significant difference in the variance (*F* test 0.01).

## 4. Discussion

The unique peculiarity of CompuFlo® compared with previous devices and those currently being tested is that the CompuFlo® system has a technology called Dynamic Pressure Sensing (DPS®) in which a maximum pressure value is set that controls the electromechanical motor of the system to limit the amount of fluid dispensed to identify the epidural space. This instrument utilizes a high resolution in-line pressure sensor that can detect pressure changes with a sensitivity of 5 *μ*V/V/mmHg, making it more sensitive than what is identified by human touch. In addition, the CompuFlo® is capable of accurately measuring the in-situ pressure with an objective pressure value. Hence, it can provide the operator with an accurate pressure measurement at the tip of the needle in real-time and on a continuous basis.

The results of our study clearly indicated that the epidural technique performed with CompuFlo® resulted, with very low variability between operators, in the needle stopping immediately after the puncture of the material that simulated the ligamentum flavum.

Instead, when the technique was performed by the operator with the traditional technique, the needle advanced considerably more forward before being stopped, and there was also extreme variability among the operators.

In a 3D reconstruction, at the most lateral parts of the epidural space, where there is no epidural fat, the ligamenta flava directly contacts the dural sac [13]; in this area, minimizing the depth of the advancement of the Tuohy needle within the epidural space is particularly crucial.

Recently, Gebhard et al. [11] performed a large, prospective, randomized, controlled, noninferiority trial to compare the CompuFlo® instrument to the standard LOR technique for lumbar epidural space identification. When examining the labor and delivery arm outcomes, they reported no cases of ADP in the Compuflo® group compared with 5.1% in the control group. Unfortunately, the ADP rate was the secondary outcome of that study, and therefore that study was not powered enough to investigate this issue. Nevertheless, this significant trend towards a reduced ADP with CompuFlo® may suggest that the high sensitivity of the instrument may help the physician to limit the extension of the Tuohy needle into the ligamentum flavum. The findings of our simulation study support this hypothesis.

We are aware of the limitations of the extrapolation of the experimental data from simulation to humans; however, materials used were identical, and participants' expertise was comparable and, in addition, a similar protocol on humans would have been impossible. We also acknowledge that, although sensible, the direct correlation between a limited extension of the Tuohy needle into the ligamentum flavum and reduced ADP rate remains to be substantiated.

In conclusion, the CompuFlo® instrument was associated to a reduced distance of the Tuohy needle tip beyond the simulated ligamentum flavum into the epidural space.

## Data Availability

The data used to support the findings of this study are available from the corresponding author upon request.
